# Single-cell regulome data analysis by SCRAT

**DOI:** 10.1093/bioinformatics/btx315

**Published:** 2017-05-12

**Authors:** Zhicheng Ji, Weiqiang Zhou, Hongkai Ji

**Affiliations:** Department of Biostatistics, Johns Hopkins University Bloomberg School of Public Health, Baltimore, MD, USA

## Abstract

**Summary:**

Emerging single-cell technologies (e.g. single-cell ATAC-seq, DNase-seq or ChIP-seq) have made it possible to assay regulome of individual cells. Single-cell regulome data are highly sparse and discrete. Analyzing such data is challenging. User-friendly software tools are still lacking. We present SCRAT, a Single-Cell Regulome Analysis Toolbox with a graphical user interface, for studying cell heterogeneity using single-cell regulome data. SCRAT can be used to conveniently summarize regulatory activities according to different features (e.g. gene sets, transcription factor binding motif sites, etc.). Using these features, users can identify cell subpopulations in a heterogeneous biological sample, infer cell identities of each subpopulation, and discover distinguishing features such as gene sets and transcription factors that show different activities among subpopulations.

**Availability and implementation:**

SCRAT is freely available at https://zhiji.shinyapps.io/scrat as an online web service and at https://github.com/zji90/SCRAT as an R package.

**Supplementary information:**

Supplementary data are available at *Bioinformatics* online.

## 1 Introduction

Single-cell regulome (scRegulome) mapping technologies such as single-cell sequencing assay of transposase-accessible chromatin (scATAC-seq) (Buenrostro *et al.*, 2015; Cusanovich *et al.*, 2015), single-cell chromatin immunoprecipitation followed by sequencing (scChIP-seq) (Rotem *et al.*, 2015) and single-cell DNase I hypersensitive site sequencing (scDNase-seq) (Jin *et al.*, 2015) have been emerging as a powerful new approach to studying gene regulation. Unlike the conventional ChIP-seq (Johnson *et al.*, 2007), DNase-seq (Crawford *et al.*, 2006) and ATAC-seq (Buenrostro *et al.*, 2013) technologies which measure average behavior of a cell population, single-cell technologies can measure regulatory element activities within each individual cell, thereby allowing one to examine the heterogeneity of a cell population. This is important for studying molecular mechanisms of tumors, immune responses, stem cell differentiation, and many other biological systems.

**Fig. 1:**
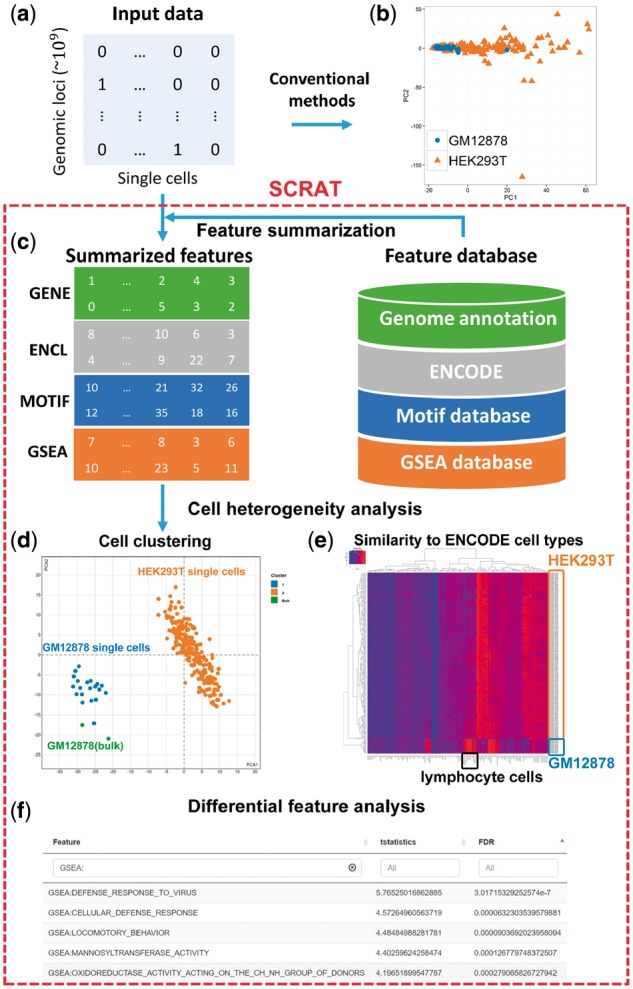
SCRAT analysis pipeline. (**a**) Single-cell regulome data is very sparse. (**b**) Analyzing scATAC-seq data using conventional bulk peak calling followed by clustering cells based on peak-level signals failed to separate two different cell types (i.e. GM12878 and HEK293T). (**c**) SCRAT first aggregates the input data into features according to empirical knowledge learned from public databases. (**d**) It then dissects cell heterogeneity by clustering cells using the aggregated features. For the same data in (b), SCRAT successfully separated GM12878 and HEK293T cells into two groups. Green dots are a few reference bulk DNase-seq samples from a precompiled database to help infer identities of cell subpopulations. (**e**) SCRAT can also evaluate the similarity between each cell and existing cell types in the precompiled database. (**f**) Finally, SCRAT identifies differential features between subpopulations of cells

Typically, a scRegulome dataset contains cells sampled from a heterogeneous cell population. Two common data analysis problems are to identify subpopulations of cells and distinguishing features that show differential regulatory signals among different subpopulations. Currently, easy-to-use software tools for these tasks are still lacking. Unlike data from the traditional bulk technologies which are relatively continuous, scRegulome data are highly sparse and discrete. For instance, chromatin accessibility measured by scATAC-seq is nearly a binary signal at each genomic locus (Fig. 1a, Supplementary Fig. S1). Using these highly sparse and discrete data to discriminate signal from noise at each individual genomic locus is extremely difficult. For this reason, conventional tools developed for analyzing bulk data are not suitable for single-cell data. Aggregating signals across multiple genomic loci with shared biological functions can mitigate sparsity and discreteness and has been shown to be a useful way to analyze scRegulome data (Buenrostro *et al.*, 2015; Cusanovich *et al.*, 2015; Rotem *et al.*, 2015) (Supplementary Material and Supplementary Fig. S2). However, systematically aggregating signals according to different genomic features (e.g. transcription factor binding motifs, gene sets) and using the aggregated signals to analyze sample heterogeneity is a non-trivial task for many investigators due to lack of software support, as demonstrated in Supplementary Tables S1 and S2. Here, we present SCRAT, a toolbox with a graphical user interface (GUI) for analyzing cell heterogeneity in single-cell regulome (i.e. scATAC-seq, scDNase-seq and scChIP-seq) data. It can be used to summarize data from each cell according to different genomic features, identify cell subpopulations based on these features, infer identities of cells in each subpopulation, and discover features that show differential regulatory signals among subpopulations (Fig. 1).

## 2 SCRAT functions and examples

The main functions of SCRAT are summarized below.

### 2.1 Data pre-processing

SCRAT takes aligned sequence reads (i.e. bam files) as input. Users have options to exclude artifact signals from the ENCODE blacklist regions (ENCODE Project Consortium, 2012) and filter out cells with low total read count.

### 2.2 Feature summarization

Next, users can aggregate reads from each cell according to different features, such as across all motif sites of each transcription factor binding motif (*Motif*), across co-regulated DNase I hypersensitive sites (DHSs) defined by ENCODE DNase-seq data (*ENCODE Cluster*), within a region of interest of each gene (*Gene*), and across all genes of each gene set in the MSigDB (Liberzon *et al.*, 2011) database (*Gene Set*). Here, motifs, DHS clusters, genes, and gene sets are called ‘features’ (Fig. 1c). For human and mouse genomes, these features are pre-defined and stored in SCRAT. Users can also define their own features for aggregation by uploading one or more lists of genomic regions in BED file format (*Custom Feature*). After aggregation, the signals for each feature are normalized to adjust for library size.

### 2.3 Cell heterogeneity analysis

SCRAT uses the aggregated signals to cluster cells into subpopulations (Fig. 1d). Multiple clustering methods are provided. Clustering can be based on one or multiple sets of features chosen by users. The cluster number may be determined automatically. One can use the original features or the transformed features after dimension reduction. Multiple dimension reduction methods are provided.

### 2.4 Inferring cell identity

Users can compare each cell’s regulome to a pre-compiled regulome database consisting of ENCODE DNase-seq profiles from a wide variety of cell types to infer the likely cell type of each cell. The similarity between each single cell and existing cell types in the database based on the aggregated signals can be visualized using a heatmap (Fig. 1e). Users can also select existing cell types in the database and project them to the principal component space of single cells to help illuminate the nature of the heterogeneity (Fig. 1d, green dots).

### 2.5 Differential feature analysis

Given cell subpopulations, users can identify features that are differential among subpopulations (i.e. heterogeneity-driving features). One can choose to run parametric (*t*-, ANOVA *F*-) or non-parametric (Wilcoxon rank-sum, Kruskal-Wallis or permutation) test on each feature to evaluate whether its aggregated signals are differential among the user-selected subpopulations. Differential features which pass certain false discovery rate cutoff will be reported (Fig. 1f).

### 2.6 GUI

SCRAT has a GUI which makes the analysis user-friendly.

Details of these functions are provided in Supplementary Material. Supplementary Table S1 compares SCRAT with existing popular tools for regulome or differential feature analyses. To demonstrate SCRAT, we analyzed a scATAC-seq dataset consisting of GM12878 and HEK293T cells (Supplementary Material). Conventional bulk peak calling followed by clustering cells using peak-level signals failed to separate the two cell types (Fig. 1b). In contrast, SCRAT successfully identified the two cell subpopulations (Fig. 1d) and differential features that matched the cell identities (Supplementary Figs. S3–S11; Supplementary Table S3). We also applied SCRAT to scATAC-seq data from human and mouse embryonic stem cells (ESC) and found that a consistent feature driving cell heterogeneity in these ESCs was cell cycle genes (Supplementary Figs. S12 and S13, Supplementary Tables S4 and S5).

In summary, SCRAT provides a set of easy-to-use tools for cell heterogeneity analysis, and it addresses the pressing needs for software support for analyzing scRegulome data.

## Supplementary Material

Supplementary Table 1

Supplementary Table 2

Supplementary Table 3

Supplementary Table 4

Supplementary Table 5

Supplementary Data
