# Supplementary material for: Single-cell regulome data analysis by SCRAT
Source: Bioinformatics. 2017 May 12;33(18):2930–2. doi: 10.1093/bioinformatics/btx315 (PMC5870556; doi:10.1093/bioinformatics/btx315)
Supplement: Supplementary Data [file btx315_supplementary_material_r1.pdf]

Supplementary Materials for

Single-cell regulome data analysis by SCRAT

Zhicheng Ji, Weiqiang Zhou and Hongkai Ji

S1. Signal aggregation helps scRegulome data analyses by transforming sparse signals into continuous features

S1.1 Single-cell regulome data are highly sparse and discrete

Unlike data from the conventional bulk technologies, data generated by single-cell regulome (scRegulome) mapping technologies are highly discrete and sparse. For example, **Supplementary Figure 1a** shows scATAC-seq data from three GM12878 single cells along with bulk ATAC-seq and bulk DNase-seq data for GM12878. The bulk DNase-seq data were obtained from the Encyclopedia of DNA Elements (ENCODE) project (ENCODE Project Consortium, 2012). Aligned reads in bam format were downloaded from http://hgdownload.cse.ucsc.edu/goldenPath/hg19/encodeDCC/wgEncodeUwDnase/. The bulk ATAC-seq data were obtained from GEO (GSM1155958) (Buenrostro *et al.*, 2013). Sequence reads in this bulk ATAC-seq dataset were aligned to human genome hg19 using bowtie (Langmead *et al.*, 2009) with parameters (-X2000 -m 1) which specify that paired reads with insertion up to 2000 base pairs (bps) were allowed to align and only uniquely aligned reads were retained. Then, PCR duplicates were removed using Picard (http://broadinstitute.github.io/picard/). The scATAC-seq data were obtained by randomly selecting three GM12878 cells from the dataset (Cusanovich *et al.*, 2015) analyzed in this article (i.e., the GM12878 data in example 1, which is described in detail in **Section S9**).

In **Supplementary Figure 1a**, the genome was divided into 200 bp non-overlapping windows, and the number of reads within each window was counted. The figure compares the window read counts across a representative genomic region between GM12878 single cells (scATAC-seq) and the bulk GM12878 samples (bulk ATAC-seq and DNase-seq). The plot clearly shows that data from scATAC-seq are highly sparse. Within each cell, most genomic windows did not have any read, and windows with reads usually only contained 1 read. By contrast, signals in the bulk ATAC-seq and DNase-seq data were much more continuous. The discreteness and sparsity of scRegulome data are not surprising for two reasons. First, each genomic locus has only up to two copies of chromatin that can be assayed within a single cell. Thus, theoretically each genomic position can contribute at most two reads to the single-cell measurements if there are no PCR duplicates. This is different from the bulk technologies in which millions of cells are pooled and analyzed together, and hence providing many more copies of chromatin for assay. Second, the total number of reads per cell produced by the current scRegulome mapping technologies is low. Take the two scATAC-seq datasets generated by (Cusanovich *et al.*, 2015) and (Buenrostro *et al.*, 2015) as an example. The average number of reads per cell for these two scATAC-seq datasets was approximately 2700 and 14000 respectively. By contrast, the bulk regulome mapping technologies typically generate tens of millions of reads per sample. It is estimated that the human genome has 10^6-10^7 regulatory elements (ENCODE Project Consortium, 2012). This means that for the scATAC-seq data above, the average read count per regulatory element within a single cell is far less than one, and most

regulatory elements do not have any read within a cell. To show it more clearly, we randomly sampled three cells from the scATAC-seq dataset generated by (Buenrostro *et al.*, 2015). These three cells represent three different cell types, GM12878, H1 and K562, respectively. We obtained DNase I hypersensitive sites (DHSs) in the human genome using the protocol described in **Section 2** ("**ENCODE cluster**") below and counted the number of reads for each DHS in each cell. **Supplementary Figure 2a-c** shows the distribution of the number of reads in individual DHSs in these three cells. The vast majority of DHSs only contained zero or one read. While some DHSs contained more than two reads (note: each DHS here is a 200 bp window instead of a single nucleotide (see **Section S2** below) and hence may have more than two reads), the number of such DHSs is very small.

Due to the sparsity of the data, accurately measuring the activity of each individual regulatory element in a single cell is difficult. Most genomic windows have less than two reads, making it extremely difficult to statistically distinguish true signals from noises. To demonstrate, **Supplementary Figure 1b** shows several genomic regions (highlighted with black, blue and red boxes). For the two regions in blue and red boxes, each region contains one read in one single cell (cell 1 and cell 3 respectively). The region in blue box has a clear signal in the bulk ATAC-seq and bulk DNase-seq samples. Thus, it is possible that the single read observed in cell 1 represents a true signal in that cell. However, since different cells within a cell population can behave differently, it is possible that the signal observed in the bulk data is present only in a subset of cells. With only one read available in cell 1, one cannot confidently rule out the possibility that this read is a random noise in that particular cell. For the region in red box, the decision is more difficult to make. This region does not have a clear signal in the bulk samples. Thus, the single read observed in cell 3 could be a random noise. However, it is also possible that this single read represents a bona fide signal present in a rare cell population (thus only a few cells including cell 3 carry the signal and most other cells do not). With only one read available, it is again difficult to distinguish between these two possibilities. Finally, for the region in black box, there is a clear peak in the bulk samples. However, this region does not contain any read in the three cells shown in the figure. Here, zero read count could mean that there is no signal in any of these three cells. It could also mean that there are signals but the scATAC-seq experiment failed to capture the signals due to the sparsity of the data. If the data are more continuous like the bulk ATAC-seq or bulk DNase-seq data, one might be able to resolve these ambiguities. Unfortunately, these ambiguities are difficult to resolve using the highly discrete and sparse data in a single cell produced by the current scRegulome mapping technologies.

S1.2 Aggregating reads from multiple related regions mitigates sparsity and discreteness

To handle the sparsity of the single-cell data, SCRAT first combines signals from multiple genomic regions that share similar biological properties and aggregates them into features. For example, one can aggregate reads across all motif sites for each transcription factor binding motif (*Motif*), across co-regulated DNase I hypersensitive sites (DHSs) defined by ENCODE DNase-seq data (*ENCODE Cluster*), across all nucleotides within a region of interest surrounding each gene (*Gene*), and across all genes of each gene set in the MSigDB database (*Gene Set*).

Take the ENCODE cluster feature as an example. An ENCODE cluster is a group of DNase I hypersensitive sites that share a similar cross-cell-type chromatin accessibility pattern in ENCODE DNase-seq data (see **Section S2** below for details). This group of DHSs can be viewed as a "pathway" consisting of co-

activated regulatory elements. For each ENCODE cluster, the read counts from all DHSs in the cluster can be added up to create an aggregated count that represents the activity of the pathway in a single cell. By aggregating reads from multiple DHSs into one feature, SCRAT transforms the sparse data into a much more continuous feature.

Below we demonstrate the advantage of signal aggregation through an analysis of differential regulatory activities between different cell types. We will show that compared to single-cell analyses based on unaggregated signals, single-cell analyses based on aggregated signals can better capture differential regulatory activities observed in the bulk data. To carry out this analysis, we obtained both bulk DNase-seq data and single-cell ATAC-seq data for three cell types GM12878, H1, and K562. The bulk DNase-seq data for GM12878, H1, and K562 were downloaded from the ENCODE project in bam format (download link: http://hgdownload.cse.ucsc.edu/goldenPath/hg19/encodeDCC/wgEncodeUwDnase/). The scATAC-seq data (Buenrostro *et al.*, 2015) for GM12878, H1 and K562 were obtained from GEO (GSE65360) and processed using the protocol described in **Section S10** below. For each cell type, one single cell was randomly sampled from the scATAC-seq dataset to conduct the analysis below. For each pair of cell types, we first calculated differential signals using the bulk DNase-seq data. We then asked how well single-cell analyses can capture differential signals observed in the bulk data.

We calculated differential signals both using the unaggregated signals at each individual DHS and using the aggregated signals for each ENCODE cluster. The DHSs were obtained using the protocol described in **Section S2** (“**ENCODE cluster**”) below. To calculate differential signals at each individual DHS, reads within each DHS were counted for each cell type. Read counts were divided by the library size of the corresponding cell type and then multiplied by a constant N (N=10,000) in order to normalize the data from different cell types. The normalized read counts were log2 transformed after adding a pseudo-count of 1. The difference in the normalized and log2-transformed read count between two cell types (i.e., the log2 fold change of the normalized read counts) was used to characterize the differential activity of each individual DHS. In order to calculate differential signals based on ENCODE clusters, we used SCRAT to group co-activated DHSs into 2000 clusters and calculated the aggregated signal for each cluster in each cell type (see **Section S2** “ENCODE cluster” for details). Data were normalized in the same way as before. Differential activity of each ENCODE cluster was then characterized by the difference in the normalized and log2-transformed signal between two cell types (i.e., log2 fold change of normalized and aggregated read count).

The results are shown in **Supplementary Figure 2d-i.** The scatter plots in **Supplementary Figure 2d-f** show the correlation between the differential signals obtained from the single-cell analysis and the differential signals from the bulk analysis when these analyses were conducted based on individual DHSs. In these plots, each dot represents a DHS. The scatter plots in **Supplementary Figure 2g-i** show the correlation between the differential signals from the single-cell and bulk analyses when the analyses were conducted based on the aggregated signals of ENCODE clusters. Here each dot in the plots represents an ENCODE cluster. These plots clearly show that the aggregated signals for ENCODE clusters are more continuous than the unaggregated signals at individual DHSs. Moreover, using the aggregated signals, single-cell analyses better captured the cell type differences observed in bulk DNase-seq data. For example, for comparing GM12878 and H1, the Pearson’s correlation between the single-cell and

bulk analyses was only 0.12 when the differential signal was studied at each individual DHS (**Supplementary Fig. 2d**). However, the correlation between the single-cell and bulk analyses increased to 0.74 when the differential signals were computed based on ENCODE clusters (**Supplementary Fig. 2g**). Similar results were obtained for comparing other cell types (**Supplementary Fig. 2e,f,h,i**).

Besides the differential analyses above, our clustering analyses in **Figure 1b,d** and **Section S9** also demonstrate that aggregated signals allow one to correctly cluster cells based on their cell type, which cannot be achieved using the unaggregated peak-level signals. Collectively, these analyses show that signal aggregation can mitigate sparsity in the scRegulome data and improve the data analysis.

S2. Feature definition

Features used by SCRAT to aggregate signals are described below.

Motif

We downloaded 1044 human and mouse transcription factor binding motifs from the JASPAR (Mathelier *et al.*, 2014) and TRANSFAC (Matys *et al.*, 2006) databases. These motifs were computationally mapped to the human (hg19, hg38) and mouse (mm9, mm10) genomes using CisGenome under its default parameter setting (Ji *et al.*, 2008). Motif sites were filtered by eliminating those without any DNase-seq read in ENCODE DNase-seq samples (using the samples mentioned in the ***ENCODE Cluster*** section below). The retained motif sites are stored in SCRAT. To calculate the aggregated signal, *x* base pair (bp) flanking region is extracted for each motif site (*x* is specified by users. By default, *x* = 100), and reads within that region are then counted. For each cell and each motif, the read counts across all motif sites are added together to produce the aggregated signal for that motif. The aggregated signals for all motifs are organized into a feature vector. Here each motif is a feature.

ENCODE Cluster

Here, each feature is a cluster of co-activated DNase I hypersensitive sites (DHSs). The aggregated signal is obtained by adding read counts across all DHSs within each cluster. DHS cluster was obtained using the procedure described below.

DNase-seq data from 123 human cell types and 56 mouse cell types were downloaded from the ENCODE project at http://hgdownload.cse.ucsc.edu/goldenPath/hg19/encodeDCC/wgEncodeUwDnase/ and http://hgdownload.cse.ucsc.edu/goldenPath/mm9/encodeDCC/wgEncodeUwDnase/. The human hg19 genome and the mouse mm9 genome were divided into 200 bp non-overlapping bins. Coordinates of these bins in other versions of genome assemblies (e.g., the human hg38 and mouse mm10 genomes) were also obtained by using the UCSC Genome Browser's *liftOver* tool (Kent *et al.*, 2002).

The number of reads falling into each bin was counted for each DNase-seq sample. To adjust for different sequencing depths, bin read counts for each sample i were first divided by the sample's total read count N_i and then scaled by multiplying a constant N ($N = \min_i\{N_i\}$, which is the minimum sample read count of all samples). After this procedure, the raw read count n_{li} for bin l and sample i was converted into a normalized read count $\tilde{n}_{li} = Nn_{li}/N_i$. The normalized read counts from replicate samples were averaged to characterize the DH level for each bin in each cell type.

Using the normalized signal, we identified putative regulatory elements in the genome. To do so, for each genome we first screened for genomic bins with normalized read count >10 in at least one cell type. We then computed a signal-to-noise ratio (SNR) for each bin in each cell type. To compute the SNR of a particular bin in a particular cell type, we collected 500 bins in the neighborhood of the bin in question and computed the average DH level of these bins. Using this average DH level as background, we computed the ratio [DH level of the bin in question + 1]/[Background DH level +1] to serve as the SNR. Genomic bins with SNR<4 in all cell types were then filtered out. The remaining bins (1,689,185 in human and 1,206,853 in mouse) are stored in SCRAT as putative regulatory elements. Each remaining bin is referred to as a DNase I hypersensitive site (DHS) in this article.

For the retained bins, the normalized DH signals were log2 transformed after adding a pseudocount 1. For each bin, the transformed signals in all cell types were then standardized to have zero mean and unit standard deviation. We then clustered these bins into 1000, 2000 and 5000 clusters using k-means clustering based on the standardized signals, and we stored the clusters in SCRAT. Here each cluster represents a group of co-activated DNase I hypersensitive sites.

To help users interpret the biological function of each cluster, we also identified the most active cell types for each cluster. For each cell type, we first calculated the average signal of all DHSs in the genome and the average signal of DHSs within each DHS cluster. Then, for each cluster and cell type, we calculated a DH enrichment score as the difference between the average signal of DHSs in the cluster and the average signal of all DHSs in the genome. According to the DH enrichment scores, for each cluster we identified the top five enriched cell types and used them to annotate the *ENCODE Cluster* features.

To aggregate signals in single-cell regulome (scRegulome) data, users first choose a cluster number. Using the stored clusters, SCRAT will then compute the aggregated read count for each cluster in each cell.

Gene

Gene annotations for human and mouse genomes were obtained from GENCODE (Harrow *et al.*, 2012) (GRCh38.p5 and GRCm38.p4) and stored in SCRAT. Users can define a region of interest within and/or around each gene. For example, one can define the region of interest to be a region from 3000bp upstream of each gene's transcription start site (TSS) to 1000bp downstream of TSS. As another example, one may also define the region of interest to be a region from 1000bp upstream of each gene's TSS to 500bp downstream of the gene's transcription end site (TES). By default, SCRAT uses the region from 3000bp upstream of TSS to 1000bp downstream of TSS as the region of interest for each gene. After the region of interest is defined, SCRAT will calculate the aggregated signal for each gene by counting the number of reads in the region of interest. The read counts from all genes are organized into a feature vector. Here each gene is a feature.

Gene Set

Gene sets were obtained from the Molecular Signature Database (MSigDB) (Liberzon *et al.*, 2011) which curates the gene sets used by the Gene Set Enrichment Analysis (GSEA) (Subramanian *et al.*, 2005).

These gene sets are stored in SCRAT. Each gene set contains a list of genes. For each gene set, SCRAT counts the total number of reads falling into the user-defined regions of interest (defined in a similar way as in the *Gene* section) of all its member genes. For each cell, the read numbers of all gene sets are organized into a feature vector. Here each gene set is a feature.

Custom Feature

There are two options for the custom feature.

First, one can upload a bed file which stores a list of genomic regions. SCRAT will count the number of reads in each region for each cell. Here each genomic region will be summarized as a feature (e.g., if the bed file contains 100 genomic regions, SCRAT will summarize 100 features).

Second, one can upload multiple bed files where each file stores a list of genomic regions. SCRAT will count the number of reads in all genomic regions of each bed file for each cell. Here each bed file will be summarized as a feature (e.g., if 100 bed files are uploaded, SCRAT will summarize 100 features).

S3. Data normalization

After obtaining the aggregated signal for each feature and each cell, SCRAT normalizes the aggregated signal to adjust for library size (i.e., total read count of each cell). Specifically, for each cell, the aggregated signal for each feature is divided by the library size of the cell and multiplied by a constant number *N* (*N*=10,000). The normalized signals are log2 transformed after adding a pseudocount 1. To characterize the variation of each feature, the coefficient of variation (i.e., the ratio between the standard deviation and the mean) is calculated and reported for each feature. Then, features that have low or constant signals across all cells are excluded. By default, a feature will be excluded if its log transformed values are less than 0.01 in more than 90% of samples or its coefficient of variation is less than 0.01.

Before subsequent analyses such as clustering, SCRAT also provides users with an option to scale the features by standardizing each feature to have zero mean and unit standard deviation.

S4. Dimension reduction

SCRAT provides two different methods to perform dimension reduction: principal component analysis (PCA) and t-Distributed Stochastic Neighbor Embedding (t-SNE) (Maaten and Hinton, 2008).

The default dimension reduction method is PCA. Let $\mathrm{E} = (\mathrm{E}_1, \cdots, \mathrm{E}_\mathrm{n})$ be a matrix containing data for H features and n cells. Each column in the matrix corresponds to a cell and each row corresponds to a feature. The matrix is standardized such that expression values within each row have zero mean and unit standard deviation. Then PCA is run on the standardized matrix, and the top K principal components (PCs) are retained. After PCA, the original E matrix is mapped to a lower dimensional space and becomes a matrix with K rows and n columns. Here K is much smaller than H.

K can be specified manually by users or automatically determined by SCRAT (**Supplementary Fig. 3a**). To determine the optimal number of K, SCRAT first calculates the variance λ_i explained by the ith PC. Let $v_i \equiv \sqrt{\lambda_i}$ be the standard deviation explained by each PC. v_i is a non-increasing function of i. SCRAT

approximates this function using a continuous piecewise linear model $v_i = \mathrm{f(i)} + \epsilon$ where ϵ represents noise and f(i) consists of two regression lines:

$$\mathrm{f(i)} = \begin{cases} \alpha_0 + \alpha_1 * \mathrm{i} & \text{if } \mathrm{i} \le \mathrm{k} \\ \beta_0 + \beta_1 * \mathrm{i} & \text{if } \mathrm{i} > \mathrm{k} \end{cases}$$

$$\text{s.t.}\, \alpha_0 + \alpha_1 * \mathrm{k} = \beta_0 + \beta_1 * \mathrm{k}$$

SCRAT computes the least squares fit of this model using the first 20 PCs or the total number of cells, whichever is smaller. The fitted model varies when one changes k. SCRAT tries different integer k and finds the k that produces the smallest squared error, $\sum_{\mathrm{i}=1}^{20}(v_i - \mathrm{f(i)})^2$. This k will be used as the optimal number of K.

The t-SNE method is originally developed as a visualization technique that helps visualizing high-dimensional data in 2-3 dimensions (Maaten and Hinton, 2008). The calculation of t-SNE is carried out using the *tsne* package (Donaldson, 2016) in R. By default, data are reduced to two dimensions and the perplexity parameter in t-SNE is set to its default value in the *tsne* package. SCRAT also provides options for users to specify the values of dimension and perplexity. In practice, we suggest users to set the dimension to 2 or 3 when using t-SNE. This is because as the t-SNE authors pointed out in their original paper, "the behavior of t-SNE when reducing data to two or three dimensions cannot readily be extrapolated to d>3 dimensions", and it is not well understood how t-SNE will perform when the data are reduced to >3 dimensions (Maaten and Hinton, 2008).

S5. Sample clustering

In order to identify cell subpopulations, SCRAT provides four methods to perform sample clustering: model-based clustering (Fraley and Raftery, 2002), hierarchical clustering, k-means clustering, and density-based clustering DBSCAN (Ester *et al.*, 1996). The default clustering method is model-based clustering.

The model-based clustering is performed using the *mclust* package (Fraley and Raftery, 2002) in R. It fits a mixture of multivariate normal distributions to the data. The variance-covariance matrix for each normal component in this mixture is designated as 'ellipsoidal, varying volume, shape and orientation'. By default, the number of clusters is chosen by *mclust* using the Bayesian Information Criterion (BIC). We also provide an option to allow users to manually specify the number of clusters. After model fitting, the posterior probability that each cell belongs to each cluster can be computed. Cells are assigned to clusters based on the largest posterior probability.

For both hierarchical clustering and k-means clustering, one can manually specify the number of clusters or use the optimal number of clusters determined by SCRAT. In order to determine the optimal number of clusters, SCRAT uses the following criterion. First, SCRAT calculates the proportion of total data variance unexplained by the clusters. More precisely, let $\mathrm{E} = (\mathrm{E}_1, \cdots, \mathrm{E}_\mathrm{n})$ be a matrix containing data for n cells. Each column in the matrix corresponds to a cell and each row corresponds to a feature (if no dimension reduction is performed) or a projection (if dimension reduction is performed). Suppose the n columns are clustered into i clusters. Let $\overline{\mathrm{E}}^\mathrm{k}$ denote the mean of the kth cluster and let $\overline{\mathrm{E}}$ be the mean of all columns. Let C(j) be the cluster membership of the jth cell. The total data variance is defined as $\mathrm{SST} = \sum_{\mathrm{j}=1}^{\mathrm{n}} ||\mathrm{E_j} - \overline{\mathrm{E}}||^2$ where $||.||$ represents l^2 norm. The variance unexplained by the cluster structure

is defined as $\mathrm{SSW} = \sum_{k=1}^{i} \sum_{j:C(j)=k} ||E_j - \overline{E}^k||^2$. The proportion of total data variance unexplained by the cluster structure is f(i)=SSW/SST. Note that f(i) is a decreasing function of i. Next, SCRAT approximates this function using a family of continuous piecewise linear models and determine the model that fits the function the best in the same way as described above to determine the optimal number of PCs (**Supplementary Fig. 3a**). The i corresponding to the best model is determined as the optimal cluster number.

The density-based clustering DBSCAN is performed using the *dbscan* package in R. This approach clusters data points based on density. It defines a data point as a core point if there are at least minPts data points within distance ε from the point in question. It then uses the core points and their density-reachable points to form clusters (Ester *et al.*, 1996). Users do not need to specify the cluster number which is implicitly determined by minPts and ε. In SCRAT, users are provided with the option to specify the ε (i.e., eps) and minPts parameters. By default, the minPts parameter (i.e., minimal number of points in the ε neighborhood to define a core point) is set to 5 as in *dbscan* package. The ε parameter is automatically chosen using the k-distance graph approach. This approach plots the *k*-nearest neighbor distances and uses the elbow point in the plot to choose the value of ε. To implement this, the kNNdist function provided by the *dbscan* package is first used to calculate k-nearest neighbors' distances for each data point in data matrix E. Here k is set to have the same value as minPts (which is suggested by the manual of the *dbscan* package). kNNdist yields a distance matrix where each row represents a data point. The matrix has k columns representing the k nearest neighbors of each data points. The matrix elements are the distances of the k-nearest neighbors to each data point. Next, the distance matrix is converted into a vector by concatenating all columns into one single column vector. Elements in this vector are then sorted and placed in an increasing order. Denote d(i) as the i-th smallest element of the ordered distance vector, then d(i) is an increasing function of i. Finally, we approximate this function using a family of continuous piecewise linear models to determine the optimal bending point i in a way similar to how we choose the optimal number of principal components in PCA and the optimal number of clusters in k-means and hierarchical clustering (**Supplementary Fig. 3b**). The d(i) corresponding to the optimal i is set to be the optimal ε. Note that the DBSCAN method can label some data points as noise (outliers) and do not assign them to any cluster. These noise points will all be labeled as cluster 0 in SCRAT. In other words, cluster 0 is a noise collector rather than a real cluster.

S6. Cell identity inference

To help users understand the nature of the heterogeneity (i.e., what cell types might be in a heterogeneous cell population), SCRAT compares each single cell with a precompiled database consisting of publicly available DNase-seq samples. To prepare the database, we first downloaded all available DNase-seq samples for both human and mouse from ENCODE. Then we applied exactly the same SCRAT protocol to all the bulk samples using default parameters described above. With the database, SCRAT calculates the pairwise Pearson's correlation between each single cell and each bulk sample in the database using the user-specified features. The resulting pairwise correlations are visualized in a heatmap (**Fig. 1e**). Note that only features that are included in both user-defined single cell analysis settings and the bulk DNase-seq database will be used to calculate the correlation.

If PCA is chosen as the method to perform dimension reduction (see **Section S4**), user can select samples from the existing cell types in the database and project them to the principal component space

generated by the scRegulome data. The selected cell types are then shown in the interactive scatterplot (**Fig. 1d, green dots**). This function is useful for illuminating properties or likely cell identities of cell subpopulations in the single cell dataset.

S7. Differential feature analysis

Given cell subpopulations, users can identify features that are differential among subpopulations. SCRAT provides both parametric and nonparametric methods to perform differential feature analysis. The parametric methods include t-test and analysis of variance (ANOVA) F-test. The nonparametric methods include Wilcoxon rank-sum test, Kruskal-Wallis test, and permutation tests based on t- and F-statistics.

If exactly two cell subpopulations (or cell clusters) are included in the differential analysis, users can choose t-test (default), Wilcoxon rank-sum test, or permutation test based on t-statistics to identify differential features. Users can specify the test to be one-sided or two-sided. For permutation test based on t-statistics, SCRAT randomly permutes the subpopulation (i.e. cluster) labels N times and recalculates the t-statistics. For each feature, a permutation p-value is computed using the N t-statistics obtained from the permutation for that feature as the empirical null distribution. The permutation p-value is calculated as the percentage of permutations that generate a t-statistic as extreme as or more extreme than the observed t-statistic. The number of permutations N is a parameter that can be specified by users (N=1000 by default). For all methods, the p-values are further adjusted using Benjamini-Hochberg (BH) procedure to obtain false discovery rate (FDR) (Benjamini and Hochberg, 1995).

If more than two subpopulations are included in the differential analysis, users can choose to perform ANOVA F-test, Kruskal-Wallis test, or permutation test based on F-statistics to identify differential features. The permutation test is conducted by permuting the subpopulation labels and computing empirical p-values as above, but replacing t-statistics by F-statistics. For all methods, p-values are adjusted using the BH procedure to obtain FDR.

S8. Comparison with other analysis tools

Supplementary Table 1 compares SCRAT with the state-of-the-art tools for analyzing regulome data or identifying differential features. First, we note that although the original studies that developed the scRegulome technologies conducted various analyses, those studies did not provide any software tool that can be directly used by other users to perform the analyses. Also, according to the website https://github.com/seandavi/awesome-single-cell which compiles software tools for single cell genomics, SCRAT is currently the only software tool available for single-cell regulome data. To perform the scRegulome analyses, users have to process a massive amount of data in order to prepare the features for aggregation. This is non-trivial. As demonstrated by **Supplementary Table 2**, SCRAT can save enormous amounts of users' time. Second, the existing ready-to-use software tools for regulome analyses are all designed for handling bulk samples. Tools for analyzing bulk samples are not suitable for analyzing the scRegulome data. For example, peak callers (e.g. MACS, CisGenome, PeakSeq, SICER, etc.) only call peaks and they do not analyze cell heterogeneity and do not address the issues induced by sparse signals. Tools for differential gene analyses, on the other hand, do not support scRegulome feature extraction (which requires aggregating signals) and identification of cell subpopulations. In

Section S9.1, we further demonstrate that bulk peak calling followed by clustering cells using peak-level signals may not be able to correctly identify cell subpopulations that can be identified by SCRAT using aggregated feature signals.

S9. Example I: Analyses of scATAC-seq data from GM12878 and HEK293T cells

We first demonstrate SCRAT by analyzing a single-cell ATAC-seq dataset (Cusanovich *et al.*, 2015) consisting of a mixture of 370 GM12878 lymphoblastoid and 344 HEK293T embryonic kidney cells. The data were obtained from GEO (GSM1647122). The paired-end reads were trimmed by Trimmomatic (Bolger *et al.*, 2014) to remove adaptor content and aligned to human genome hg19 using bowtie2 (Langmead and Salzberg, 2012) with parameter -X2000 which specify that paired reads with insertion up to 2000 bp were allowed to align. PCR duplicates were removed using Picard (http://broadinstitute.github.io/picard/). The aligned reads were then assigned to individual cells based on the barcode information. SCRAT allows users to exclude artifact signals from the blacklist regions defined by ENCODE (https://sites.google.com/site/anshulkundaje/projects/blacklists). Data from these regions are usually artifacts. Blacklist regions were excluded from this analysis. We also excluded cells with total read count < 500. From the remaining cells, we randomly sampled 230 HEK293T cells and 20 GM12878 cells to mimic a situation where a heterogeneous cell sample contains a relatively rare cell subpopulation. Below, we demonstrate SCRAT using this dataset.

S9.1 Conventional regulome analysis methods failed in analyzing the single cell regulome data

Before we demonstrate SCRAT, we first illustrate why the conventional bulk regulome analysis tools are not suitable for analyzing scRegulome data. The conventional regulome analysis usually begins with peak calling. We applied the popular peak caller MACS (Zhang *et al.*, 2008) to identify peaks from our data. Since scATAC-seq data are very sparse and discrete (each cell has only thousands of reads, as compared to tens of millions of reads a typical bulk sample would have) and existing peak callers are all designed for bulk samples with more continuous signals, we first pooled all single cells together and then run peak calling on the pooled sample. A total of 23,493 peaks were obtained (q-value < 0.05). For each peak region, we then extracted its read count in each single cell. Since traditional peak callers do not provide this read extraction function, this was done using SCRAT (by providing peaks as "Custom Features" for aggregation). The extracted read counts were then normalized across cells as in SCRAT. Note that the peak read counts were very sparse due to the low total read count of each single cell. These normalized peak-level read counts were then used for analyzing cell heterogeneity.

The conventional peak callers do not provide functions to aggregate signals across multiple genomic loci, clustering cells or performing heterogeneity analysis. Therefore, we used the SCRAT protocol to cluster cells using the peak-level signals obtained above. **Supplementary Figure 4a** shows the cells based on the first two principal components of peak-level signals. The GM12878 cells and HEK293T cells overlapped and cannot be separated. Applying the model-based clustering after PCA dimension reduction similar to SCRAT yielded 4 clusters which failed to separate the two cell types (clusters 1, 2 and 3 contained cells from both cell types) (**Supplementary Fig. 4b**). We also tried to cluster the cells using other methods and failed to separate the two cell types. For example, **Supplementary Figure 4c** shows hierarchical clustering of cells based on the peak-level signals without PCA dimension reduction. The GM12878 cells

(red) and HEK293T cells (blue) were mixed together. Thus, using conventional peak calling followed by cell clustering cannot correctly identify the two cell subpopulations which is the basis for cell heterogeneity analysis.

S9.2 Demonstration of SCRAT analysis

Next, we demonstrate major functions of SCRAT using the same dataset. SCRAT was able to correctly separate the two cell subpopulations and dissect the cell heterogeneity. Details about the various function menus used here are provided in the *User Manual of SCRAT* at the following Github website https://github.com/zji90/SCRATdata/blob/master/manual.pdf. The aligned bam files (aligned to hg19) for this example are available at https://github.com/zji90/SCRATdata/tree/master/SCRAT_example_data_bam. In order to help users conveniently test SCRAT, this dataset can be directly loaded into SCRAT by using the "*Load example data*" function in Step 1 (see **Supplementary Fig. 6c** below).

For readers' convenience, we also saved the SCRAT summarized features (obtained after performing Step 1 and Step 2 below) of this dataset and provide them at the following web link: https://github.com/zji90/SCRATdata/blob/master/SCRAT_summarized_features_GM12878_HEK293T.txt. If users start the SCRAT analysis from these summarized features, they can skip the procedure described below in Step 1 and Step 2, and use instead the "*Upload Summarization Table*" function in Step 2 to read in the summarized features (**Supplementary Fig. 5a**). For instance, one can upload the data in the "*Input Summary Table*" section using the "*Choose Files*" button, and read in the data using the "*Read in*" button after the upload is completed (**Supplementary Fig. 5b**). The summarized features can be viewed in the "*Results*" panel (**Supplementary Fig. 5d**). Then, one can proceed to Step 3 and Step 4. To help users conveniently test SCRAT, we also allow users to directly load the summarized features of this example dataset into SCRAT by clicking the "*Load example summarized features*" button in Step 2 (**Supplementary Fig. 5c**). After loading these summarized features, one can then proceed to Step 3 and Step 4.

Step 1: Data input and preprocessing

The first step is to input the single-cell data (aligned bam files) into SCRAT. First, one has to select the corresponding reference genome from the "*Select Genome*" section (**Supplementary Fig. 6a**). Then, one can upload bam files in the "*Input Bam Files*" section using the "*Choose Files*" button, and read in the data using the "*Read in*" button after the upload is completed (**Supplementary Fig. 6b**). To help users test SCRAT, the bam files for this example can be directly loaded into SCRAT by clicking the "*Load example data*" button (**Supplementary Fig. 6c**). By default, SCRAT will filter blacklist regions and exclude samples with total number of reads less than 500 (adjustable by the user). Information about the input data will be shown after they have been read in (**Supplementary Fig. 6d**). One can proceed to feature summarization using the "*Next step*" button (**Supplementary Fig. 6e**).

Step 2: Feature summarization

The second step is to summarize the input data into different features according to the feature definitions. To demonstrate, we summarized signals in this test dataset using the pre-defined features in

SCRAT. In the "*Choose Summarizing Method*" section, we selected all pre-defined SCRAT features for our analysis (i.e., *Motif*, *ENCODE Cluster*, *Gene* and *Gene set*; **Supplementary Fig. 7a**).

SCRAT provides rich tuning options for each feature type. The parameters of each feature types can be adjusted in the "*Method Details*" section of the user interface (**Supplementary Fig. 7d**). When the example dataset was summarized based on *Motif*, we asked SCRAT to aggregate reads within 100 base pair (bp) flanking region from both sides of the motif sites. When the data were summarized based on *ENCODE Cluster*, we set the cluster number to be 2000. When the data were summarized based on *Gene*, we asked SCRAT to aggregate reads within the 3000 bp upstream and 1000 bp downstream region from the transcription start site (TSS) of each gene. When the data were summarized based on *Gene Set*, we chose to include only Gene Ontology (GO) gene sets for analysis. For each gene set, we asked SCRAT to aggregate reads within the 3000 bp upstream and 1000 bp downstream regions from TSSs of all genes.

SCRAT allows one to normalize the features and filter them based on the user-provided parameters (**Supplementary Fig. 7b**; also see **Section S3**). Once all parameters are set, one can start the summarization process using the "*Run Summarization*" button (**Supplementary Fig. 7c**). After the summarization is done, the summarized features can be viewed and downloaded from the "*Results*" panel (**Supplementary Fig. 7e**). Then, one can proceed to cell heterogeneity analysis using the "*Next step*" button (**Supplementary Fig. 7f**).

Step 3: Cell heterogeneity analysis

The third step is to dissect the cell heterogeneity by clustering the cells. First, one can select different types of features for clustering in the "*Select Feature Type*" section (**Supplementary Fig. 8a**). Second, one can choose what type of methods to use to reduce the dimension of the features in the "*Dimension reduction method*" section (**Supplementary Fig. 8b**). Third, one can choose the clustering method in the "*Clustering method*" section (**Supplementary Fig. 8c**). By default, SCRAT selects the *ENCODE Cluster* features and uses the principal components of these features to cluster cells based on model-based clustering. One can start the clustering process using the "*Perform Clustering*" button (**Supplementary Fig. 8d**). Then, the result will be shown in the "*Clustering Result*" panel (**Supplementary Fig. 8e**). For example, we applied this default procedure to the example data as shown in **Supplementary Figure 8**. In other words, we used co-activated DHS clusters (i.e., *ENCODE cluster*) to aggregate signals. As described before, the cluster number of the ENCODE cluster feature was set to 2000 in Step 2. This means that DHSs in the human genome were grouped into 2000 clusters based on their co-activation patterns observed in the ENCODE DNase-seq data. For each DHS cluster, SCRAT added up the read counts of all DHSs in the cluster in each cell. The aggregated and normalized read count was used to represent the overall activity of the cluster in that cell. In this way, the scATAC-seq data in each cell were summarized into 2000 features. Our clustering used these 2000 features as input. Before clustering, we reduced the feature dimension using PCA, and the number of PCs was automatically determined by SCRAT. We then clustered cells using model-based clustering, and the cluster number was also automatically determined by SCRAT. This produced the results shown in **Figure 1d** and **Supplementary Figure 8e**. Cells were correctly grouped into two clusters, corresponding to GM12878 and HEK293T respectively.

After obtaining the cell clustering results, one can further explore the cell identities by comparing the individual cells with the existing cell types in our pre-compiled bulk DNase-seq database. First, one can use the "*Include existing cell types*" function (**Supplementary Fig. 8f**, **Fig. 1d**) to select samples from the existing cell types in the database and project them to the principal component space of the single cells. Second, one can also evaluate the similarity between each cell and the existing cell types (**Fig. 1e, Supplementary Fig. 10**) using the "*Similarity to existing cell types*" function (**Supplementary Fig. 8g**) based on the selected features (**Supplementary Fig. 9a**). One can start the analysis using the "*Calculate Correlations*" button (**Supplementary Fig. 9b**). The results will be visualized as a heatmap (**Supplementary Fig. 9c**).

In this test dataset, these analyses (**Fig. 1d-e, Supplementary Fig. 10**) correctly identified that the rare subpopulation of cells (GM12878, lymphoblastoid) were closely related to lymphocyte cell types (e.g., GM12878, GM12864 and GM12865).

Then, one can proceed to differential feature analysis using the "*Next step*" button (**Supplementary Fig. 8h**).

Step 4: Differential feature analysis

The last step is to identify the differential features among different cell subpopulations (**Fig. 1f**). One can perform analysis to all cell clusters obtained from Step 3 or a subset of selected cell clusters (**Supplementary Fig. 11a**). Then, one can choose a statistical test (**Supplementary Fig. 11b**). If more than two cell clusters are selected, ANOVA F-test, Kruskal-Wallis test, or permutation test based on F-statistics can be used to identify differential features. If only two cell clusters are selected, t-test, Wilcoxon rank-sum test, or permutation test based on t-statistics can be used. One can click the "*Perform Test*" button to start the analysis (**Supplementary Fig. 11c**). The results including the name of the feature, the test statistics and the adjusted p-value (FDR) will be shown in the "*Results*" panel (**Supplementary Fig. 11d**). For example, we analyzed the *Gene Set* features in our test data using t-test. Differential gene sets between the two major subpopulations (GM12878 and HEK293T) were identified and sorted based on false discovery rate (FDR<0.05). GO gene sets that distinguished the two subpopulations included "defense response to virus" and "cellular defense response" which were enriched in GM12878 cells, and "vasculature development" and "organ morphogenesis" which were enriched in HEK293T cells. For the *Motif* features, motifs that distinguished the two subpopulations included IRF1 and STAT1 which were enriched in GM12878 cells. These results matched well with the distinct biology of the two underlying cell types (**Supplementary Table 3**).

S10. Example 2: SCRAT analysis of human and mouse embryonic stem cells

As a second example, we applied SCRAT to analyze single-cell ATAC-seq data from 96 human embryonic stem cells (H1-hESCs) and 96 mouse embryonic stem cells (mESCs) (Buenrostro *et al.*, 2015). These data were obtained from GEO (GSE65360). For each cell, paired-end reads were trimmed using the program provided by Buenrostro *et al.* (Buenrostro *et al.*, 2015) to remove adaptor content and aligned to the corresponding genome (human genome hg19 for H1-hESCs or mouse genome mm10 for mESCs) using bowtie2 with parameter -X2000. PCR duplicates were removed using Picard.

We first analyzed the single-cell ATAC-seq dataset for 96 human embryonic stem cells (H1-hESCs). After excluding reads from the blacklist regions, 95 cells with ≥500 reads were retained for subsequent analyses. These cells were then clustered using the *ENCODE Cluster* features (model-based clustering after PCA dimension reduction; the optimal number of PCs and the cluster number were both automatically determined). This resulted in 4 clusters (**Supplementary Fig. 12a**). The cluster 4 contained only one outlier cell which could be either contaminating cells or rare cells with distinct properties. Excluding this outlier cluster, we asked what features are driving the heterogeneity among the remaining cell clusters. We performed differential feature analysis using clusters 1, 2 and 3 (ANOVA F-test) and ranked the differential features based on FDR (FDR<0.05 were considered as significant). The top ranked gene sets contained gene sets related to cell cycles such as "mitotic cell cycle checkpoint" and "G1 phase". Among these, the "mitotic cell cycle checkpoint" gene set ranked at top 1 (**Supplementary Fig. 13a**).

Interestingly, when we applied the same analysis to the single-cell ATAC-seq data for 96 mouse embryonic stem cells (mESCs) (all 96 cells were retained for analysis), a similar pattern was found. The mESCs were grouped into 3 clusters (**Supplementary Fig. 12b**). The "mitotic cell cycle checkpoint" gene set was also ranked as the top 1 gene set feature driving the heterogeneity of these cells (**Supplementary Fig. 13b**). The full differential feature analysis results for hESCs and mESCs can be found in **Supplementary Tables 4-5**. The fact that mitotic cell cycle checkpoint genes were found to be the top gene set in the analyses of both human and mouse indicates that a major factor driving the heterogeneity of ESCs is likely related to cell cycle.

S11. SCRAT installation

SCRAT can be used online. SCRAT online GUI can be launched directly at https://zhiji.shinyapps.io/scrat/. Users only need a web browser to use SCRAT online and no additional software is required. Reference genomes supported by the SCRAT online GUI include hg19, hg38, mm9 and mm10.

SCRAT can also be installed locally on users' computer via Github. If users choose to do so, they should install R on their computer before installing SCRAT. R can be downloaded at http://www.r-project.org/ (R version 3.2.5 and up is recommended). Users should first install the SCRAT data packages by running the following commands in R:

```
if (!require("devtools"))
install.packages("devtools")
devtools::install_github("SCRATdatahg19","zji90")
devtools::install_github("SCRATdatahg38","zji90")
devtools::install_github("SCRATdatamm10","zji90")
devtools::install_github("SCRATdatamm9","zji90")
```

Then, one can install the latest version of SCRAT via Github by running the following commands in R:

```
source("https://raw.githubusercontent.com/zji90/SCRATdata/master/installcode.R")
```

If one wants to test the local version of SCRAT using the data in example 1, one should install the example data package using the following commands in R:

```
if (!require("devtools"))
install.packages("devtools")
devtools::install_github("SCRATexample","zji90")
```

Finally, one can launch the user interface of SCRAT by running the following commands in R:

```
library(SCRAT)
SCRATui()
```

A graphical user interface will appear. The GUI is the same as the web interface of the online version of SCRAT. The example dataset can be then loaded into SCRAT by using the "*Load example data*" function in Step 1.

Reference

Benjamini,Y. and Hochberg,Y. (1995) Controlling the false discovery rate: a practical and powerful approach to multiple testing. *Journal of the royal statistical society.Series B (Methodological)*, **57**, 289-300.

Bolger,A.M. *et al*. (2014) Trimmomatic: a flexible trimmer for Illumina sequence data. *Bioinformatics*, **30**, 2114-2120.

Buenrostro,J.D. *et al*. (2013) Transposition of native chromatin for fast and sensitive epigenomic profiling of open chromatin, DNA-binding proteins and nucleosome position. *Nature methods*, **10**, 1213-1218.

Buenrostro,J.D. *et al*. (2015) Single-cell chromatin accessibility reveals principles of regulatory variation. *Nature*, **523**, 486-490.

Cusanovich,D.A. *et al*. (2015) Multiplex single-cell profiling of chromatin accessibility by combinatorial cellular indexing. *Science*, **348**, 910-914.

Donaldson,J. (2016) tsne: T-Distributed Stochastic Neighbor Embedding for R (t-SNE). *R package version 0.1-3*.

ENCODE Project Consortium. (2012) An integrated encyclopedia of DNA elements in the human genome. *Nature*, **489**, 57-74.

Ester,M. *et al*. (1996) A density-based algorithm for discovering clusters in large spatial databases with noise. **, 96**, 226-231.

Fraley,C. and Raftery,A.E. (2002) Model-based clustering, discriminant analysis, and density estimation. *Journal of the American statistical Association*, **97**, 611-631.

Harrow,J. *et al*. (2012) GENCODE: the reference human genome annotation for The ENCODE Project. *Genome Res.*, **22**, 1760-1774.

Ji,H. *et al*. (2008) An integrated software system for analyzing ChIP-chip and ChIP-seq data. *Nat.Biotechnol.*, **26**, 1293-1300.

Kent,W.J. *et al*. (2002) The human genome browser at UCSC. *Genome Res.*, **12**, 996-1006.

Langmead,B. and Salzberg,S.L. (2012) Fast gapped-read alignment with Bowtie 2. *Nature methods*, **9**, 357-359.

Langmead,B. *et al*. (2009) Ultrafast and memory-efficient alignment of short DNA sequences to the human genome. *Genome Biol.*, **10**, R25.

Liberzon,A. *et al*. (2011) Molecular signatures database (MSigDB) 3.0. *Bioinformatics*, **27**, 1739-1740.

Maaten,L.v.d. and Hinton,G. (2008) Visualizing data using t-SNE. *Journal of Machine Learning Research*, **9**, 2579-2605.

Mathelier,A. *et al*. (2014) JASPAR 2014: an extensively expanded and updated open-access database of transcription factor binding profiles. *Nucleic Acids Res.*, **42**, D142-7.

Matys,V. *et al*. (2006) TRANSFAC and its module TRANSCompel: transcriptional gene regulation in eukaryotes. *Nucleic Acids Res.*, **34**, D108-10.

Subramanian,A. *et al*. (2005) Gene set enrichment analysis: a knowledge-based approach for interpreting genome-wide expression profiles. *Proc.Natl.Acad.Sci.U.S.A.*, **102**, 15545-15550.

Zhang,Y. *et al*. (2008) Model-based analysis of ChIP-Seq (MACS). *Genome Biol.*, **9**, R137-2008-9-9-r137. Epub 2008 Sep 17.

Supplementary Figures

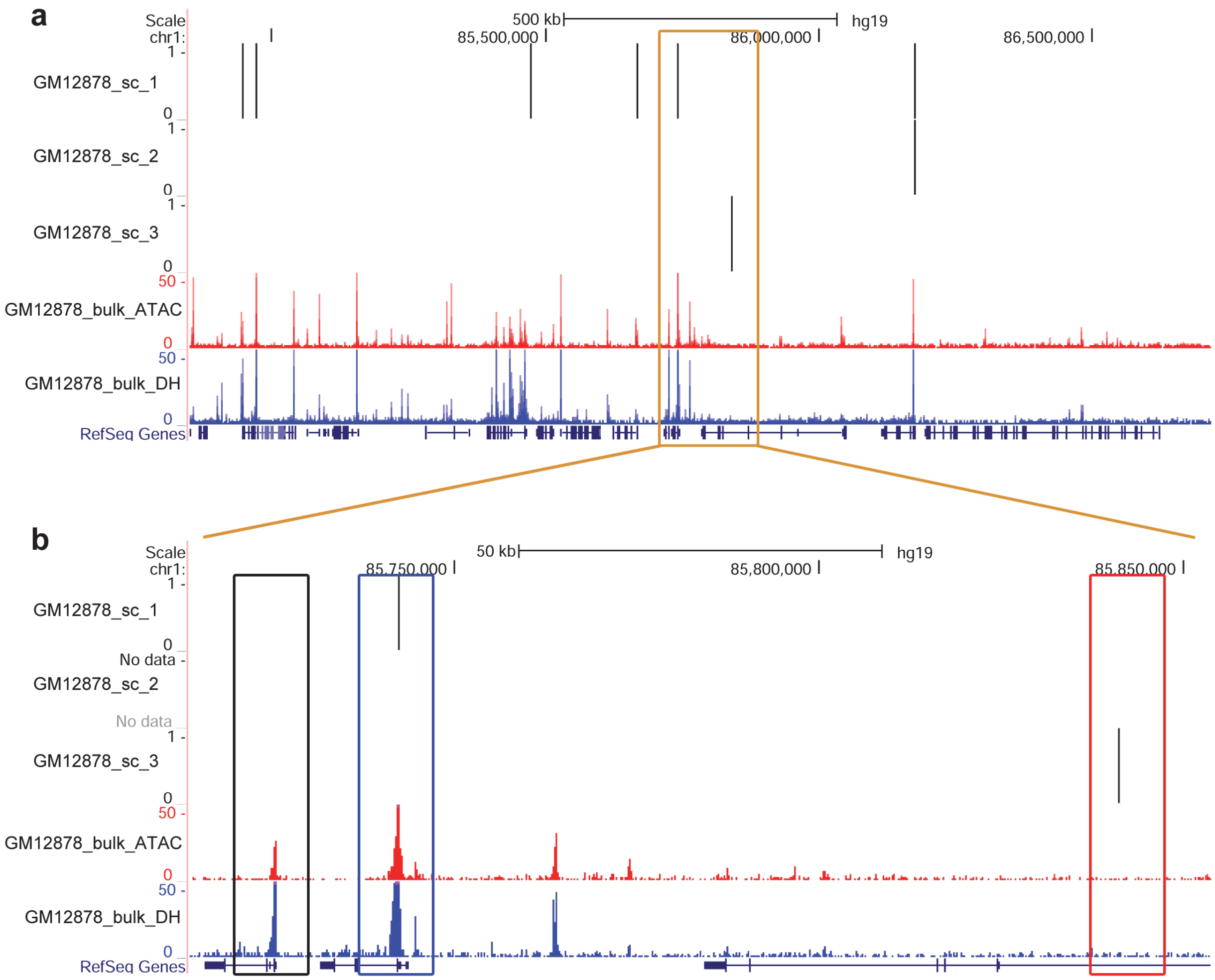

Supplementary Figure 1. Illustration of the sparsity of single-cell regulome data. (**a**) Comparison of signals from single-cell ATAC-seq, bulk ATAC-seq and bulk DNase-seq for GM12878 in a representative genomic region. Genome was divided into 200bp non-overlapping windows. For each track, the plot shows read counts in all 200bp non-overlapping windows in this genomic region. GM12878_bulk_ATAC: bulk ATAC-seq sample; GM12878_bulk_DH: bulk DNase-seq sample; GM12878_sc_1, GM12878_sc_2, and GM12878_sc_3: single-cell ATAC-seq from three different cells. (**b**) Zoom-in view of a genomic region in **a**. The black, blue and red boxes highlight three regions. The region in blue box contains a single read in GM12878_sc_1. The region in red box contains a single read in GM12878_sc_3. The region in black box contains no read in the three single cells.

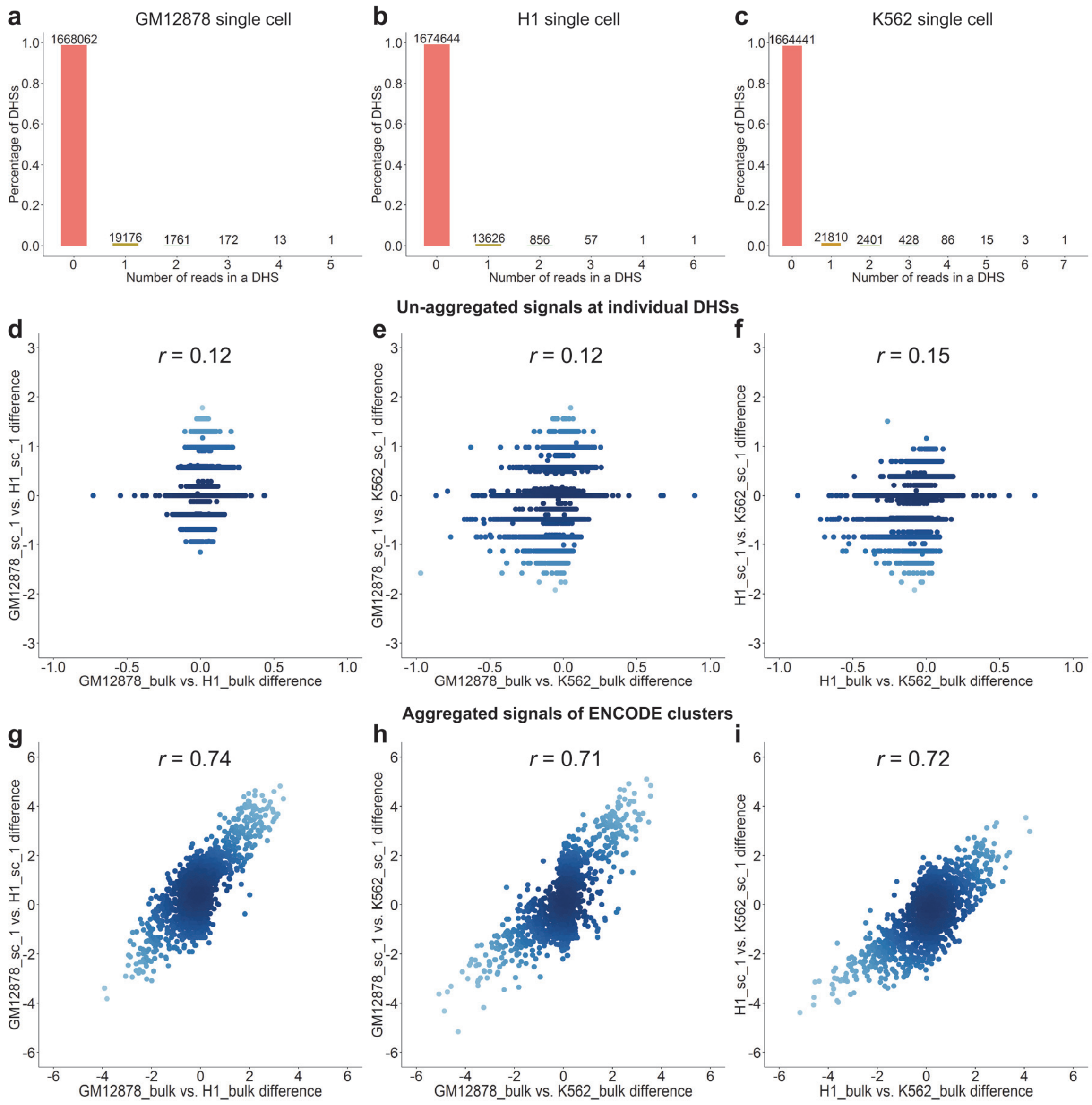

Supplementary Figure 2. Signal aggregation mitigates sparsity and discreteness.

(**a**)-(**c**) Distribution of the number of scATAC-seq reads in individual DHSs in three single cells. These three cells were from GM12878 (**a**), H1 (**b**) and K562 (**c**) respectively. Y-axis shows the percentage of DHSs in each category. The number above each bar shows the number of DHSs in each category.

(**d**)-(**i**) Comparison of the unaggregated signals of individual DHSs and the aggregated signals of ENCODE clusters in terms of their ability to capturing differential chromatin accessibility between cell types. The scatter plots show the correlation between the differential signals (log2 fold change) obtained from the single-cell analysis and the differential signals obtained from the bulk analysis (here the bulk analysis is used as a gold standard). "r": Pearson correlation.

(**d**)-(**f**) Differential signals between GM12878 and H1 (**d**), GM12878 and K562 (**e**), and H1 and K562 (**f**) were analyzed using the unaggregated signals at each individual DHS. Each dot represents a DHS.

(**g**)-(**i**) Differential signals between GM12878 and H1 (**g**), GM12878 and K562 (**h**), and H1 and K562 (**i**) were analyzed using the aggregated signals for each ENCODE cluster. Each dot represents a ENCODE cluster.

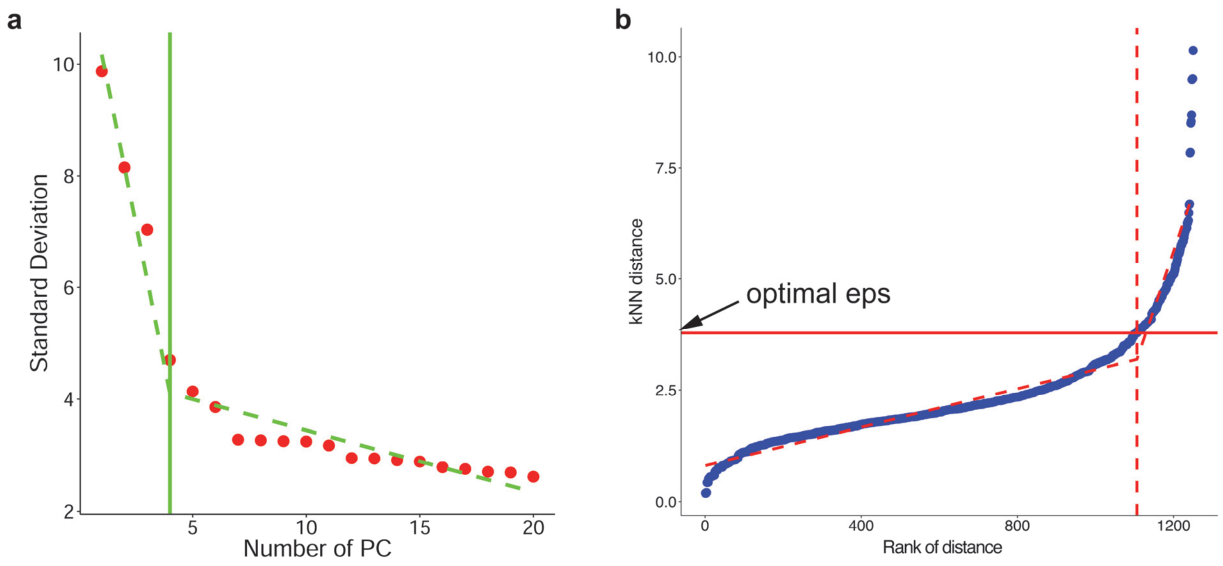

Supplementary Figure 3. Illustration of the methods used by SCRAT to automatically choose the optimal number of principal components in PCA (**a**) and the optimal value of the epsilon (ϵ) parameter in DBSCAN clustering (**b**).

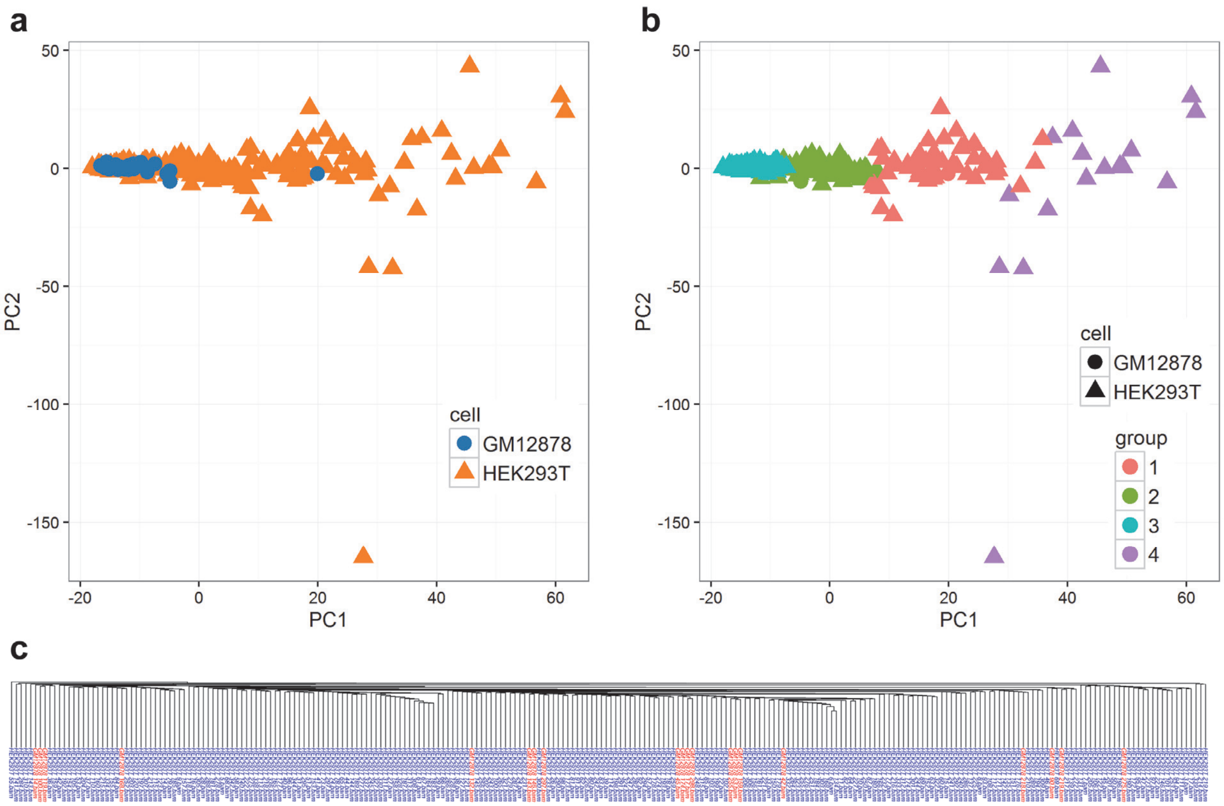

Supplementary Figure 4. Using conventional methods to analyze the single-cell regulome data. (**a**) Cells are shown using the first two principal components of the peak-level signals. The colors show cells' true identities (blue: GM12878; orange: HEK293T). (**b**) Model-based clustering grouped cells into 4 clusters which cannot separate the GM12878 and HEK293T cells. (**c**) Hierarchical clustering using the peak-level signal also failed to separate GM12878 and HEK293T cells (red: GM12878; blue: HEK293T).

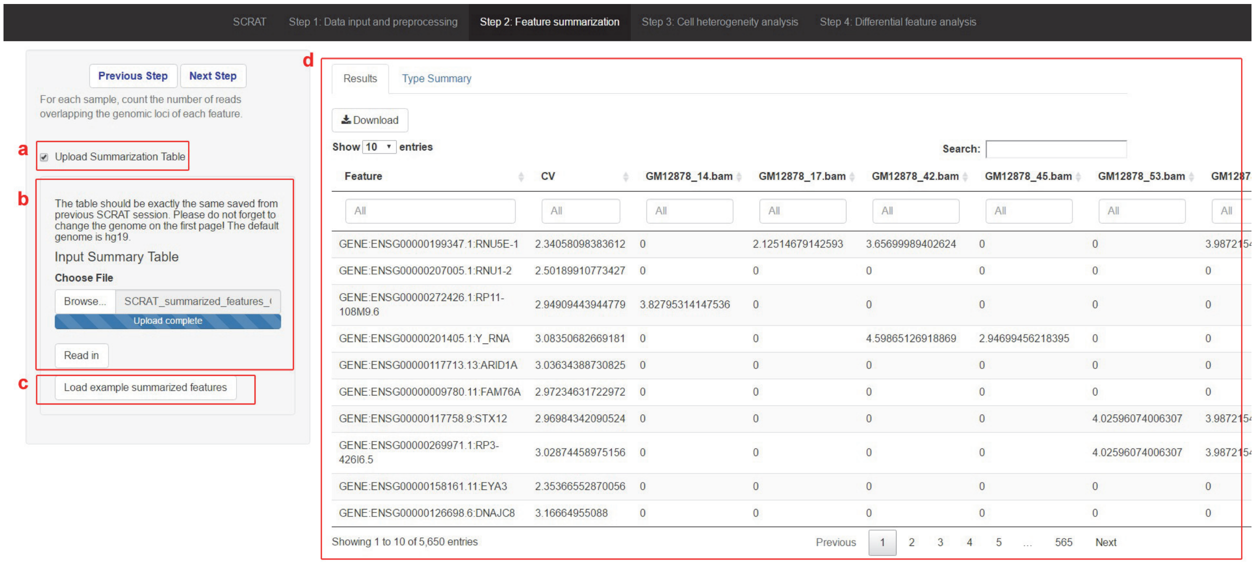

Supplementary Figure 5. User can upload the previously saved summarized features into SCRAT for analysis.

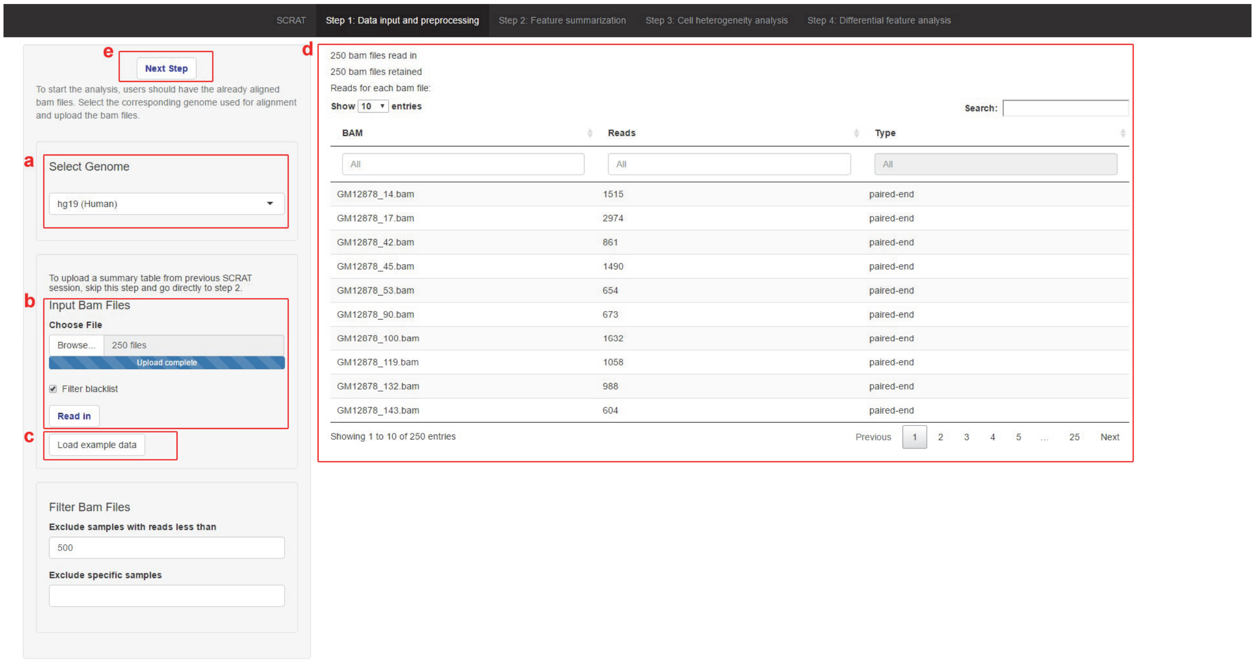

Supplementary Figure 6. SCRAT analysis step 1 -- Data input and preprocessing.

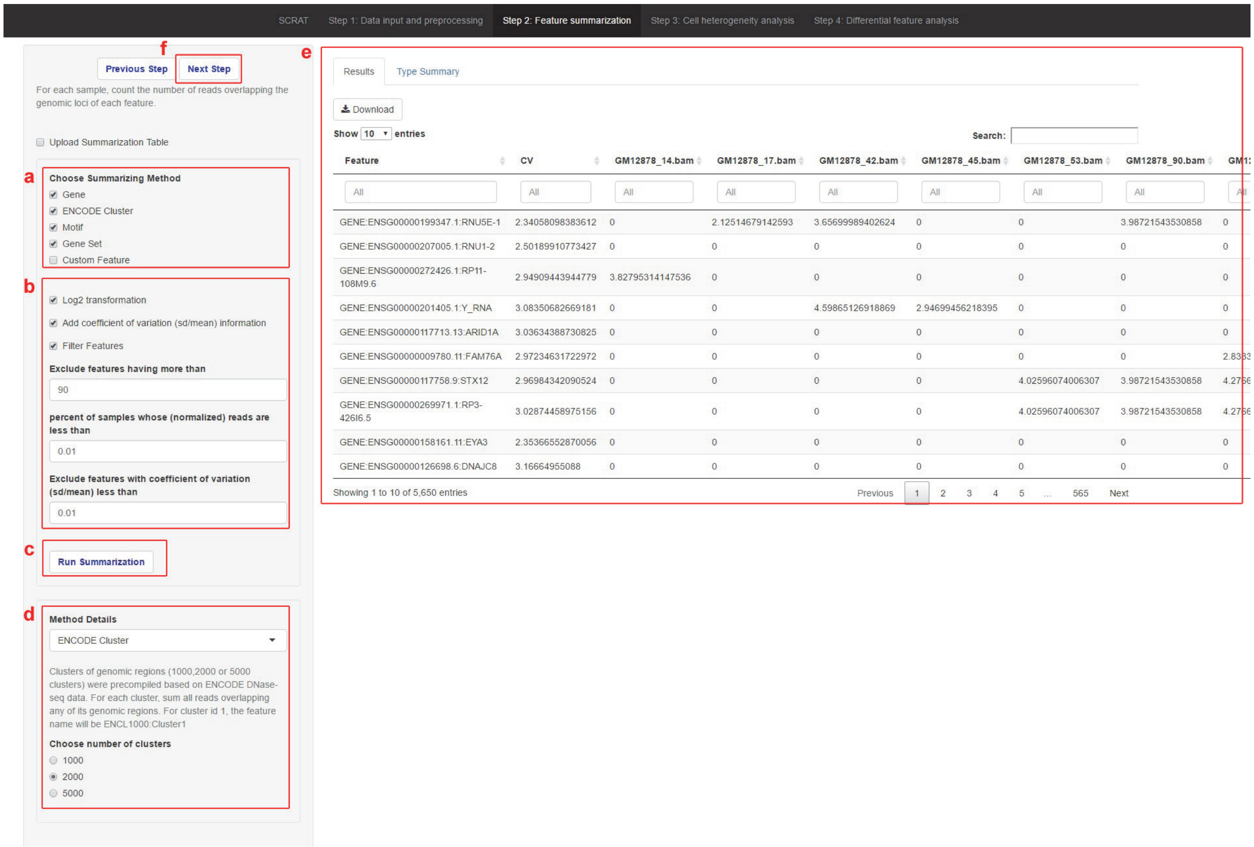

Supplementary Figure 7. SCRAT analysis step 2 -- Feature summarization.

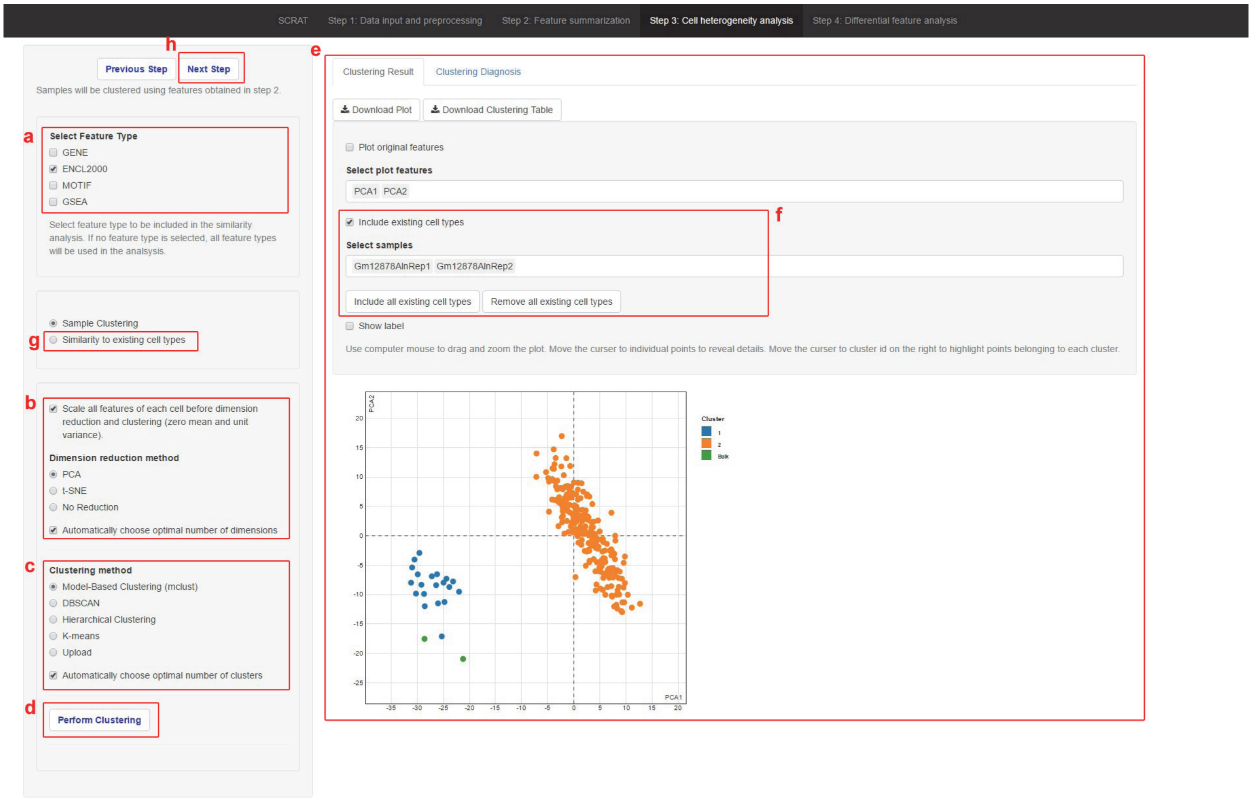

Supplementary Figure 8. SCRAT analysis step 3 -- Cell heterogeneity analysis.

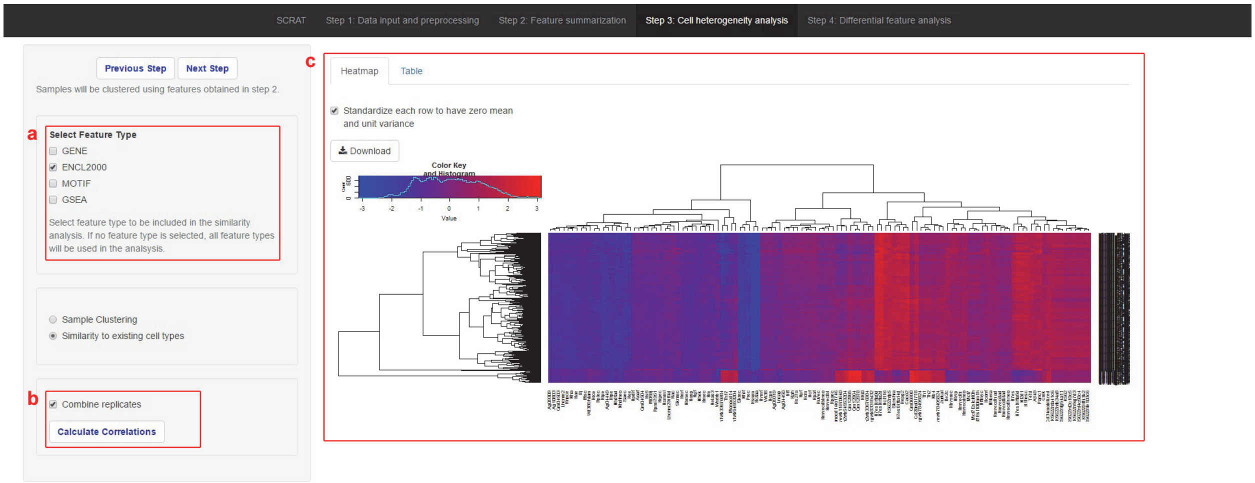

Supplementary Figure 9. SCRAT analysis step 3 -- Cell heterogeneity analysis (cont'd). Evaluating similarity to existing cell types.

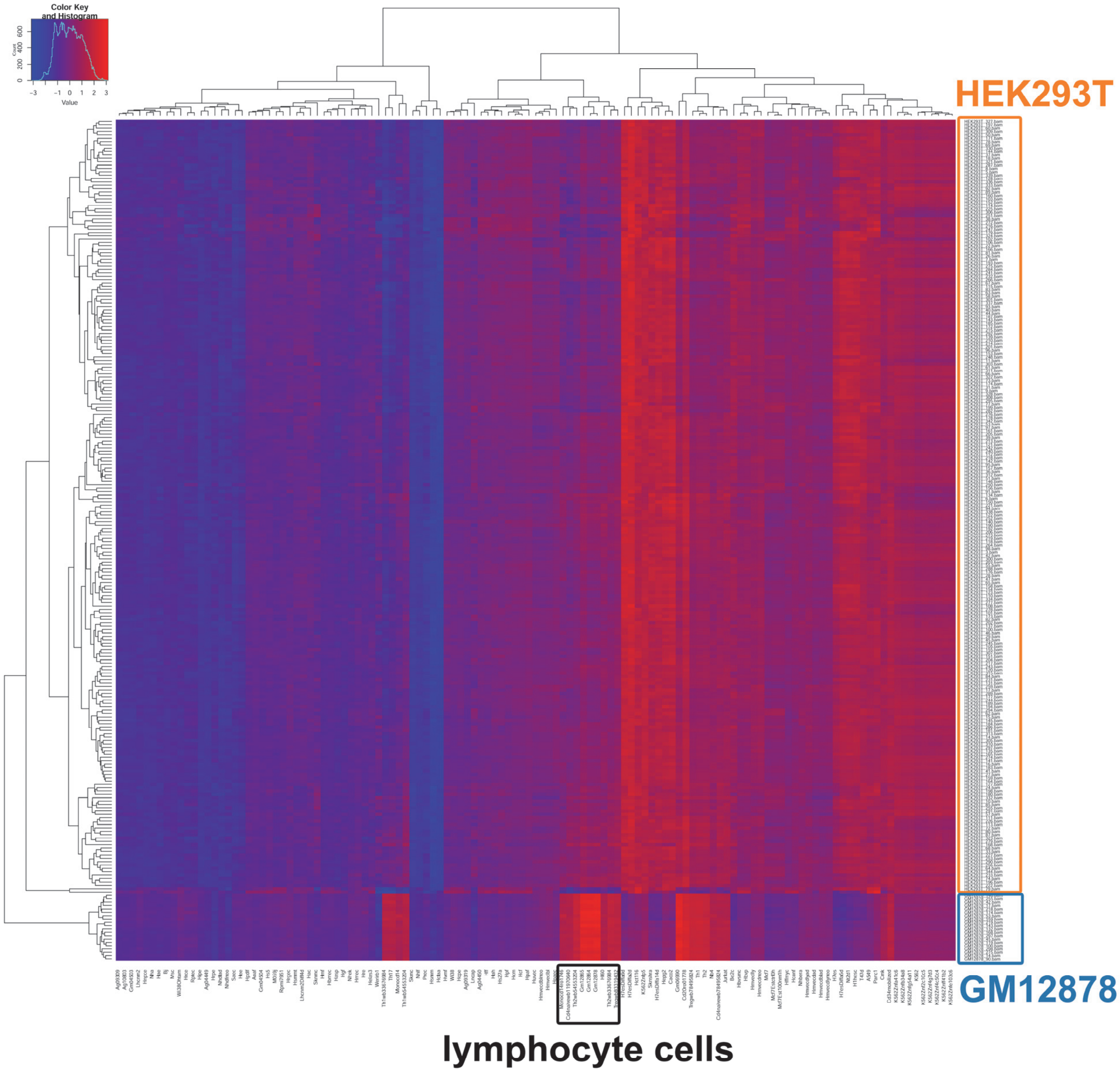

Supplementary Figure 10. Similarities between the single cells (GM12878 and HEK293T) and the existing cell types in example 1. The similarity in this figure is measured by Pearson's correlation between each single cell and each existing cell type based on the *ENCODE Cluster* features. Rows correspond to single cells and columns correspond to existing cell types. Colors in the heatmap reflect the standardized correlation (each row is standardized to have zero mean and unit variance).

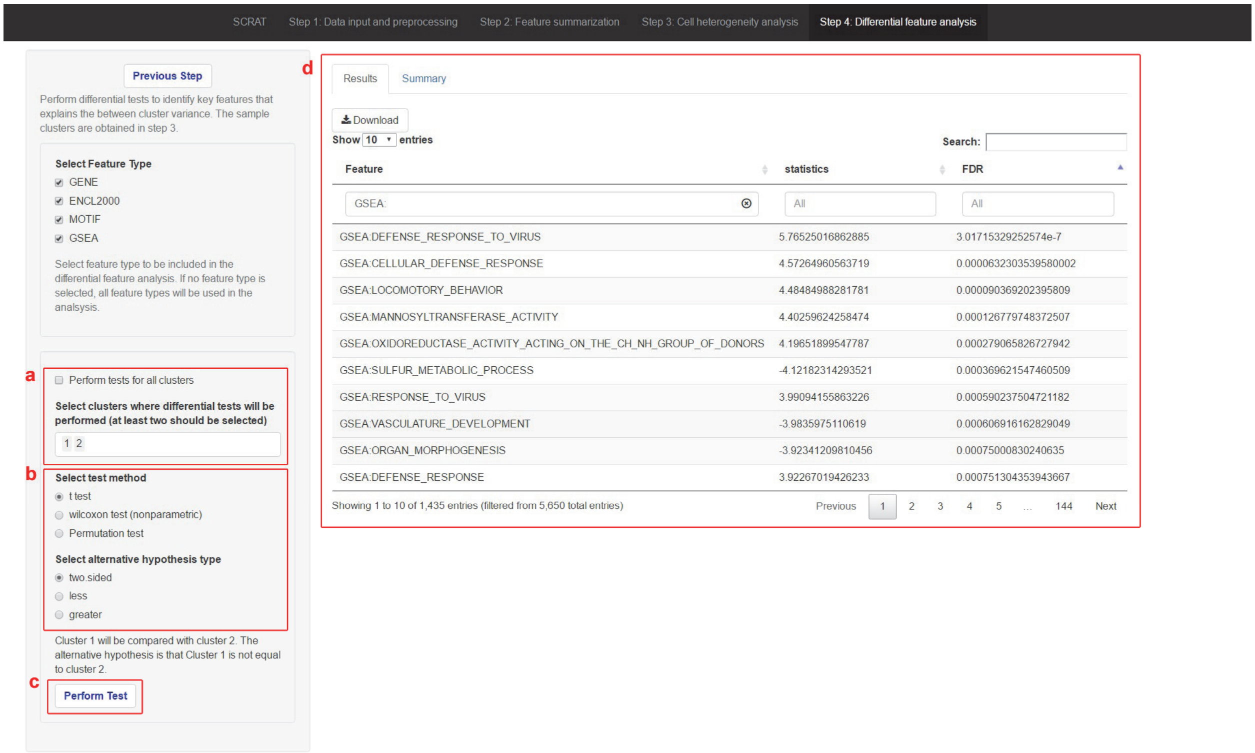

Supplementary Figure 11. SCRAT analysis step 4 -- Differential feature analysis.

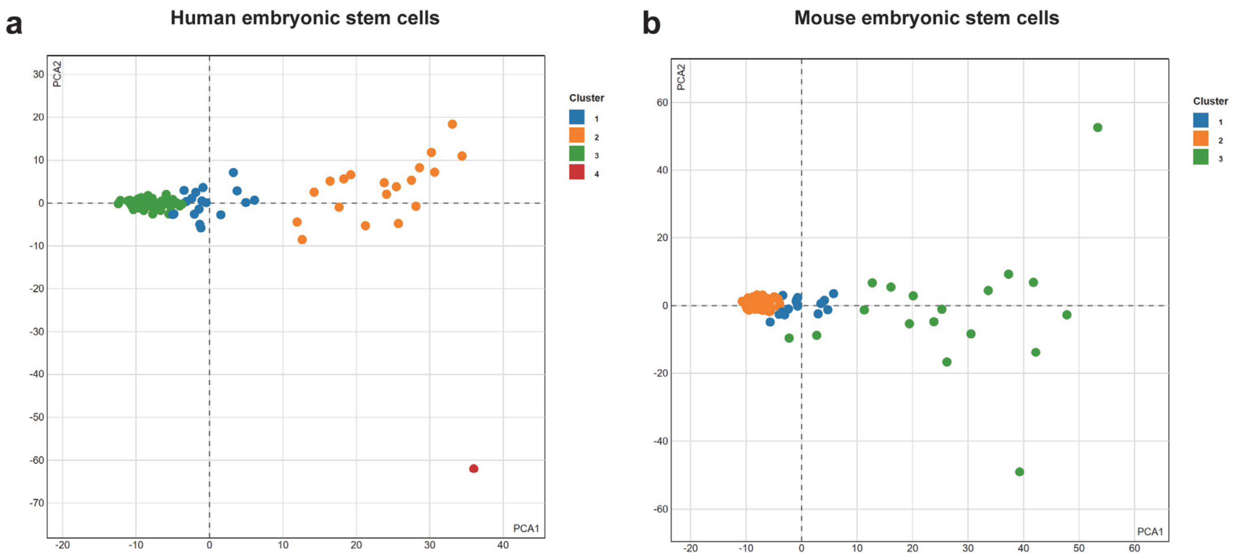

Supplementary Figure 12. Cell heterogeneity analysis for the (**a**) human and (**b**) mouse embryonic stem cells. The first two principal components from the *ENCODE Cluster* features were shown. Cell clusters obtained by SCRAT were marked with different colors.

a

Human embryonic stem cells

Feature	Fstatistics	FDR
GSEA:	All	All
GSEA:MITOTIC_CELL_CYCLE_CHECKPOINT	68.1641476075421	6.2298100359503e-16
GSEA:REGULATION_OF_CELLULAR_COMPONENT_SIZE	61.9384750832229	3.49904232441553e-15
GSEA:CALCIUM_ION_TRANSMEMBRANE_TRANSPORTER_ACTIVITY	58.343335364834	1.01411018851994e-14
GSEA:CARBOHYDRATE_BINDING	57.8632724400133	1.19521209038192e-14
GSEA:REGULATION_OF_ACTIN_FILAMENT_LENGTH	57.0601478019488	1.45339719353187e-14
GSEA:STRIATED_MUSCLE_DEVELOPMENT	56.049020714961	2.02949460975257e-14
GSEA:MEMBRANE_FUSION	55.1051653855547	2.60155640129854e-14
GSEA:G1_PHASE	54.5752375615731	3.07699975919005e-14
GSEA:SYNAPSE	52.9841010351754	5.18212504321118e-14
GSEA:SKELETAL_MUSCLE_DEVELOPMENT	52.5672627955498	6.03171004696311e-14

Showing 1 to 10 of 1,454 entries (filtered from 21,527 total entries) Previous 1 2 3 4 5 … 146 Next

b

Mouse embryonic stem cells

Feature	Fstatistics	FDR
GSEA:	All	All
GSEA:MITOTIC_CELL_CYCLE_CHECKPOINT	69.9316542751036	4.93233664928972e-16
GSEA:ALCOHOL_METABOLIC_PROCESS	65.5328893122011	2.2609156335548e-15
GSEA:ATPASE_ACTIVITY_COUPLED_TO_TRANSMEMBRANE_MOVEMENT_OF_IONS	64.9112278514366	2.76586866609048e-15
GSEA:SMALL_GTPASE_REGULATOR_ACTIVITY	63.9699274952787	3.59627076387042e-15
GSEA:PROTEIN_SERINE_THREONINE_PHOSPHATASE_ACTIVITY	62.5411685267579	5.41255859102247e-15
GSEA:SYNAPSE	60.9587988778977	9.53653341770164e-15
GSEA:DI__TRI_VALENT_INORGANIC_CATION_TRANSPORT	59.2922999998953	1.57804336372472e-14
GSEA:RAS_GTPASE_ACTIVATOR_ACTIVITY	59.109973937858	1.66233568098839e-14
GSEA:CALCIUM_CHANNEL_ACTIVITY	58.4727859922905	2.08664938815235e-14
GSEA:VIRAL_REPRODUCTION	57.9317950837411	2.34482109487198e-14

Showing 1 to 10 of 1,454 entries (filtered from 22,016 total entries) Previous 1 2 3 4 5 … 146 Next

Supplementary Figure 13. Top ranked gene sets for differential feature analysis from (**a**) human and (**b**) mouse embryonic stem cells.

Supplementary Tables

Supplementary Table 1. Comparison of SCRAT with existing popular software tools for regulome or differential feature analyses

(see Supplementary_table_1.xlsx)

Supplementary Table 2. Burden required to build features for aggregating signals in scRegulome analyses

(see Supplementary_table_2.xlsx)

Supplementary Table 3. T-statistic and FDR from the differential feature analysis of the GM12878 and HEK293T cells

(see Supplementary_table_3.xlsx)

Supplementary Table 4. F-statistic and FDR from the differential feature analysis of the human embryonic stem cells

(see Supplementary_table_4.xlsx)

Supplementary Table 5. F-statistic and FDR from the differential feature analysis of the mouse embryonic stem cells

(see Supplementary_table_5.xlsx)
